# DNA Mixture Deconvolution: A Four-Strategy Framework from Physical Separation to Database Searching

**DOI:** 10.3390/genes17040434

**Published:** 2026-04-09

**Authors:** Qiang Zhu, Zhigang Mao, Ji Zhang

**Affiliations:** 1 West China School of Basic Medical Sciences & Forensic Medicine, Sichuan University, Chengdu 610041, China; zhuqiang@scu.edu.cn; 2 Department of Laboratory Medicine, West China Hospital, Sichuan University, Chengdu 610041, China

**Keywords:** DNA mixture, deconvolution, single-cell isolation, microhaplotype, probabilistic genotyping, database searching

## Abstract

DNA mixture interpretation remains one of the most technically demanding challenges in forensic genetics. While probabilistic genotyping (PG) systems have substantially advanced likelihood ratio (LR) evaluation, comparatively less attention has been devoted to the systematic reconstruction of contributor genotypes, particularly in no-suspect and database-search contexts. This review synthesizes recent developments in DNA mixture deconvolution through a four-strategy framework: (i) physical and biological separation, (ii) high-information genetic markers, (iii) continuous probabilistic algorithms, and (iv) integration with database searching infrastructures. Upstream approaches, including single-cell isolation and sequencing, reduce mixture complexity at the molecular level. Marker innovations such as microhaplotypes, MiniHaps and DIP-STRs increase per-locus information content and enhance resistance to degradation. Downstream probabilistic models—extended from STRs to SNPs and microhaplotypes—leverage quantitative signal data to infer contributor genotypes, with recent advances in Hamiltonian Monte Carlo, variational inference, and deep learning improving inferential stability and reconstruction accuracy. Importantly, genotype deconvolution and LR evaluation represent mathematically distinct objectives, requiring different validation metrics and potentially separate architectural optimization. The convergence of molecular innovation, algorithmic refinement, and LR-based database searching is progressively transforming mixture interpretation from a purely evidential assessment into an integrated investigative framework. Future progress will depend on standardized marker panels, deconvolution-specific performance metrics, and scalable LR-enabled database infrastructures.

## 1. Introduction

DNA mixtures remain among the most analytically challenging forms of forensic biological evidence. Unlike single-source profiles, mixed DNA samples require inference of multiple contributors’ genotypes whose signals often overlap and are subject to stochastic effects such as allele drop-out, drop-in, peak height imbalance and amplification variability [1,2]. These effects are particularly pronounced in low-template and trace samples, where random sampling during PCR can distort observed profiles relative to true genotypes.

Mixture interpretation generally addresses two related but distinct questions. The first concerns evaluation of whether a specific individual contributed to the mixture, typically through likelihood ratio (LR)–based approaches. The second involves reconstruction of contributor genotypes when no suspect profile is available. While extensive methodological development has focused on LR calculation and validation, genotype reconstruction itself has received relatively limited synthesis across molecular and computational developments. This distinction is operationally important: deconvolution primarily serves investigative objectives, tolerates different error structures, and requires different validation metrics than evidential LR reporting.

Existing reviews have largely centered on LR-based probabilistic genotyping (PG) systems, including comparisons of EuroForMix, DNAStatistX, and STRmix™ [3], foundational descriptions of STRmix™ [4], and national validation frameworks such as the NIST Scientific Foundation Review [5]. In contrast, the logically prior task of genotype reconstruction—particularly in no-suspect contexts—has not been comprehensively synthesized across molecular and computational developments.

**Figure 1:**
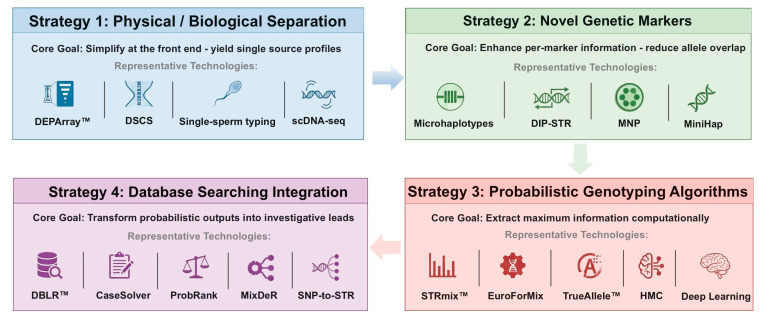
**Conceptual four-strategy framework for DNA mixture deconvolution.** Strategies are organized by analytical layer rather than importance. Physical and biological separation (Strategy 1) reduces or removes mixture complexity at cellular level when feasible. High-information genetic markers (Strategy 2) decrease allele sharing and stochastic interference at the molecular level. PG algorithms (Strategy 3) model quantitative signal data to infer contributor genotypes. Database integration (Strategy 4) translates deconvolved outputs into investigative leads. Downstream strategies address residual complexity when upstream simplification is limited or impractical. Representative technologies and performance indicators are shown for each layer.

This review addresses that gap by organizing advances in DNA mixture deconvolution into a four-strategy framework (Figure 1): 1. Physical and biological separation (Section 2); 2. High-information genetic markers (Section 3); 3. Probabilistic algorithms (Section 4); 4. Database integration (Section 5). The strategies are presented in order of analytical depth: upstream approaches reduce mixture complexity at the molecular level, while downstream approaches extract maximal information from residual overlap.

The strategies are organized by analytical layer—from molecular to computational—rather than by importance or recommended order of application. In practice, the choice of strategy depends on sample characteristics, available instrumentation, and investigative context.

This review adopts a narrative, expert-based approach and does not follow a systematic review protocol. Literature was identified through searches of PubMed, Web of Science, and Google Scholar using keywords including “DNA mixture,” “deconvolution,” “probabilistic genotyping,” “micro-haplotype,” “single-cell forensic,” and “database searching.” The search covered publications through early 2026, with emphasis on developments from 2018 onward. Additional references were identified through citation tracking and expert knowledge. Inclusion was guided by relevance to genotype reconstruction rather than LR-focused evaluation, consistent with the stated scope of this review.

## 2. Physical and Biological Separation: Simplification at the Analytical Front End

Physical separation of individual contributor cells prior to DNA amplification represents the most direct method of reducing mixture complexity. When successful, computational deconvolution is no longer required.

### 2.1. Single-Cell and Micro-Scale Separation

Several micro-scale and single-cell separation technologies have been developed to isolate individual contributor cells prior to DNA amplification, each leveraging different physical or biological properties for cell selection.

Several recent reviews have synthesized the current state and future potential of single-cell approaches in forensic applications [6,7].

Laser capture microdissection (LCM) represents an earlier-generation approach to cell-type-specific isolation, enabling targeted recovery of spermatozoa or other cell populations from tissue sections under microscopic guidance. Although LCM has demonstrated proof-of-concept utility in forensic research, its practical adoption has been limited by labor-intensive workflows, low throughput, variable DNA recovery, and the need for specialized equipment [8].

Fluorescence-activated cell sorting (FACS) offers an alternative separation modality based on fluorescent labeling of cell-type-specific surface markers. A recent study demonstrated successful FACS-based isolation of sperm and vaginal epithelial cells, enabling single-source STR profiling from mixed sexual assault samples [9].

Single-cell approaches are particularly advantageous for mixtures involving related individuals, where shared allelic profiles between contributors and non-donor relatives can lead to false inclusions under conventional bulk analysis and probabilistic genotyping frameworks [10].

#### 2.1.1. DEPArray™ Digital Microfluidics

DEPArray™ employs dielectrophoresis to isolate individual cells based on morphology and surface markers (e.g., CD45 for leukocytes) [8]. In comparative sexual assault studies, single-source STR profiles were recovered from sperm cells in 96% of samples, compared with 32% using conventional differential extraction [11]. By isolating cells prior to amplification, cross-contributor allele overlap is eliminated at the source.

#### 2.1.2. Direct Single-Cell Subsampling (DSCS)

DSCS collects multiple single cells or “mini-mixtures” containing a small number of cells (typically ~2–5 per reaction), each amplified independently [12,13]. By reducing allele overlap within each reaction, PG systems can more reliably infer contributor genotypes. In laboratory two-person mixtures at a 1:50 ratio, DSCS combined with STRmix™ or EuroForMix increased minor contributor LRs from approximately 10^1^–10^2^ to approximately 10^11^ [12,14].

#### 2.1.3. Single Sperm Typing and Clustering

In multi-suspect sexual assault cases, dozens to hundreds of individual sperm cells can be STR-typed as haploid profiles [15,16]. Clustering algorithms integrated with STRmix™ (e.g., FaSTR™) group sperm from the same male donor to reconstruct diploid genotypes for database searching [16]. This strategy has resolved mixtures otherwise intractable by bulk analysis.

### 2.2. Single-Cell Sequencing

Single-cell DNA sequencing (scDNA-seq) and chromatin accessibility sequencing (scATAC-seq) enable genome-wide SNP capture from thousands of individual cells without prior cell-type targeting [6,17]. Computational clustering separates contributors based on SNP similarity while inferring sex and ancestry. Empirical five-person mixtures and simulated eleven-person mixtures have been resolved using such approaches [10]. Unlike targeted isolation, single-cell sequencing treats contributor separation as a high-dimensional clustering problem rather than marker-based filtering.

### 2.3. Applicability and Constraints

Physical separation offers maximal interpretive simplicity by generating single-source genetic profiles. However, applicability is limited to well-preserved, cell-rich samples. Costs, instrumentation, and workflow complexity currently restrict routine deployment. Accordingly, physical separation should be viewed as a selective but highly effective upstream strategy.

Several practical constraints limit the applicability of physical separation approaches. A single diploid human cell contains approximately 6 pg of DNA, often necessitating whole-genome amplification (WGA) prior to STR typing, which introduces allelic imbalance, dropout, and drop-in artifacts [18]. Cell degradation—particularly in aged casework samples, touch DNA deposits, and environmentally exposed material—reduces cell membrane integrity and increases the risk of DNA loss during isolation [8,11]. The DEPArray™ efficiency data reported above (96% vs. 32%) were obtained under controlled laboratory conditions using well-preserved samples; casework materials with substantial degradation are expected to yield lower recovery rates [12]. Rapid DNA instruments (e.g., ANDE, RapidHIT ID) have streamlined single-source reference processing but are currently not designed for mixture interpretation, as their analytical pipelines lack the probabilistic modeling required for multi-contributor deconvolution [19].

Complementary molecular approaches—including DNA methylation-based tissue identification [20] and mRNA body fluid profiling [21]—can provide valuable contextual information about the cellular composition of mixtures, even though they do not directly reconstruct individual genotypes. For example, differential methylation patterns can confirm the presence of semen, blood, or saliva, informing analyst decisions about contributor number (NOC) specification and guiding the selection of appropriate separation or computational strategies.

## 3. Novel Genetic Markers: Information Enhancement

When physical separation is impractical, increasing per-locus information content provides an alternative strategy for reducing deconvolution ambiguity.

Table 1 provides a systematic overview of six of the marker types discussed in Section 3.1, Section 3.2, Section 3.3, Section 3.4, Section 3.5, Section 3.6 and Section 3.7 for DNA mixture deconvolution. For each marker, the table lists core molecular characteristics, deconvolution advantages, known limitations, the maximum validated mixture complexity, optimal application scenarios, and representative technology platforms. The subsections that follow discuss each marker category in detail.

### 3.1. Traditional STR Systems: Capabilities and Limitations

Conventional capillary electrophoresis STR (CE-STR) analysis relies heavily on peak height ratios for contributor inference. In complex mixtures, allele sharing—where different individuals possess alleles of identical length—reduces locus-level discriminative power [33,34]. Stutter artifacts, amplification imbalance, and degradation-induced allele drop-out further complicate interpretation [35,36].

Beyond autosomal STRs, Y-chromosome STRs remain the principal complementary marker in sexual assault casework, enabling recovery of male haplotypes even at female-to-male ratios exceeding 1000:1 [37]. The ISFG DNA Commission has issued dedicated interpretation guidelines [38], and rapidly mutating Y-STRs (mutation rates > 10^−2^) enhance discrimination among paternally related males [39]. Several countries have incorporated Y-STR profiles into national offender databases [40]. However, Y-STRs identify paternal lineages rather than reconstruct individual autosomal genotypes. Although earlier concerns about linkage and locus non-independence have been substantially addressed through dedicated statistical frameworks [38], Y-STR profiling remains complementary to, rather than a substitute for, autosomal genotype-level deconvolution. Accordingly, this review focuses on markers capable of full genotype-level deconvolution, while recognizing Y-STRs as valuable front-line triage tools.

Similarly, mitochondrial DNA (mtDNA) analysis provides lineage-level information complementary to autosomal genotyping. NGS-based sequencing of the mitochondrial genome enables quantitative detection of minor mtDNA contributors through variant allele frequency analysis, substantially improving mixture resolution compared with Sanger sequencing [41,42]. However, nuclear insertions of mitochondrial DNA (NUMTs) represent a recognized analytical confound, as co-amplified NUMT sequences can introduce phantom variants that may be misinterpreted as minor contributor signals or heteroplasmy [43,44]. Bioinformatic strategies for NUMT detection and filtering are under active development [45]. Like Y-STRs, mtDNA identifies maternal lineages rather than individual autosomal genotypes and thus serves a complementary role in the deconvolution framework presented here.

### 3.2. NGS-STR: Sequence Polymorphism in Traditional Loci

NGS-based STR typing distinguishes length-identical alleles by internal sequence variation, increasing observable alleles by approximately 23–30% depending on population and locus [22,23,24,25]. Shorter amplicons (<150 bp) improve degraded DNA performance [46,47]. However, sequence-based stutter persists, and data analysis is computationally more demanding than CE-based workflows [22,48]. Additionally, differential amplification efficiency among length-variant alleles introduces allele balance distortions distinct from traditional CE-based stutter patterns, requiring adapted analytical thresholds in downstream probabilistic analysis. Compared with CE-STR artifacts (electrophoretic stutter, pull-up, off-scale peaks), NGS-STR data exhibit unique noise sources including PCR duplicate reads, sequencing errors particularly in homopolymeric regions, and strand bias—artifacts that require marker-specific noise models rather than simple threshold-based filtering.

### 3.3. SNP-Based Markers

#### 3.3.1. Identity-Informative SNPs (iiSNPs)

SNPs have been used for human identification in forensic genetics for over two decades, with large panels developed specifically for individual discrimination. Identity-informative SNPs (iiSNPs), when combined with autosomal STRs, yield combined match probabilities of 10^−73^–10^−79^ [49,50]; variation in flanking regions can further reduce match probability by approximately 2175-fold [51]. Large iiSNP panels (e.g., QIAGEN 140-plex) support mixture deconvolution within PG frameworks, particularly when genotypes of major contributors are known [47,49].

#### 3.3.2. Forensic Investigative Genetic Genealogy (FIGG) SNPs and MixDeR Tool

When DNA databases provide no direct hits, Forensic Investigative Genetic Genealogy (FIGG) can generate investigative leads through kinship matching. However, FIGG SNP chips (containing hundreds of thousands of markers) generally assume single-source input. The MixDeR tool is specifically designed for deconvolving FIGG mixture data [52]. This open-source R package with a Shiny interface processes ForenSeq Kintelligence^®^ SNP genotyping results (containing thousands of genealogy-related SNPs) and performs deconvolution using EuroForMix. MixDeR then filters outputs to produce inferred single-source genotypes formatted for GEDmatch^®^ PRO [52,53]. Testing demonstrated successful deconvolution of two-person mixtures at ratios up to 1:20, enhancing the investigative utility of mixed samples for FIGG applications [52].

#### 3.3.3. A Prospective Framework: Bridging SNP Deconvolution with STR Databases

The following discussion outlines a conceptual framework that has not yet been empirically implemented or validated as an integrated workflow. As whole-genome sequencing (WGS) and high-density SNP panels become increasingly accessible in forensic laboratories [54,55,56,57,58,59], an important translational question emerges: whether deconvolved SNP profiles from mixtures can be linked to existing STR-based criminal databases. Genetic record-matching studies have established that linkage disequilibrium between genome-wide SNPs and STR loci can connect individuals typed on non-overlapping marker sets, with accuracy reaching 90–100% in single-source record-matching studies (not yet validated for post-deconvolution imputation), depending on panel size and population match [60,61,62,63].

Meanwhile, SNP mixture deconvolution continues to mature through tools such as MixDeR [52] and clustering-based approaches applied to NGS-derived read frequencies [64]. Together, these developments suggest a potential framework in which SNP-based deconvolution and STR imputation operate sequentially: contributors are first separated at the SNP level, and inferred genotypes are subsequently mapped onto STR loci for database comparison. Such integration could expand the utility of WGS-derived SNP data and provide investigative entry points for samples that fail conventional STR typing.

However, this paradigm remains prospective. STR imputation accuracy is sensitive to ancestry mismatches between sample and reference panel, errors introduced during deconvolution would propagate through the imputation, and the admissibility of imputed genotypes has not been tested in court. No end-to-end workflow integrating SNP deconvolution with STR imputation has yet been empirically validated. At present, the approach is best regarded as a conceptual framework rather than an evidentially validated solution.

### 3.4. Microhaplotypes (MHs): Optimal Markers for Mixture Analysis

#### 3.4.1. Definition and Core Advantages

MHs comprise tightly linked SNPs (typically 2–6) within short (<300 bp) amplicons [65,66]. MHs provide several advantages for mixture analysis. First, they reduce allele sharing: 96.4% of MHs alleles in two-person mixtures represent contributor-specific haplotype combinations, compared with 51.3% for CE-STR alleles [26]. With effective allele number (Ae) >3.0, over 95% of mixtures can be detected using only five loci [67]. Second, short amplicons (<300 bp) confer improved resistance to DNA degradation [68]. Third, sequencing read depth enables more precise contributor ratio estimation with minimal stutter and balanced amplification [26,69]. A head-to-head comparison of MHs and STR panels on identical two- and three-contributor mixtures reported 4–5× lower per-locus genotype error rates for MH-based deconvolution [27]. Fourth, a 163-plex panel successfully resolved 2–5 person mixtures [28].

It is worth noting that multi-nucleotide polymorphism (MNP) markers—combining 2–5 SNPs within ultra-short fragments (<75 bp)—represent a specialized subset of the MH concept optimized for severely degraded DNA where standard MH amplicons (<300 bp) may still fail [70,71,72].

#### 3.4.2. Locus Design Principles and Standardization

The Microhaplotype Working Group of the ISFG has proposed locus selection criteria including Ae ≥3, minimum allele frequency ≥0.05, cross-population validation, and amplicon length <300 bp [73]. Several validated MH panels (74-, 87-, 124-, and 163-plex) provide a foundation for laboratory implementation [28].

### 3.5. DIP-STR: Allele-Specific Amplification Markers

DIP-STR combines deletion/insertion polymorphisms (DIPs) with adjacent STR loci and employs allele-specific amplification. Primers are designed to selectively amplify one DIP allele together with its associated STR, enabling preferential amplification of minor contributor DNA during PCR [30,32]. This approach enables detection of minor contributors in highly unbalanced two-person mixtures (up to 1:1000) [32]. A 10-plex DIP-STR panel can selectively amplify 0.03–0.1 ng of minor DNA in the presence of a 1000-fold excess of major DNA [30,31]. DIP-STR systems are particularly suited to sexual assault and trace DNA scenarios.

### 3.6. Mini-Haplotypes (MiniHaps): Ultra-High Information Markers

In practice, the key operational distinction between MHs and MiniHaps is the phasing requirement: standard MHs containing 2–4 SNPs can be reliably phased from short-read sequencing data, whereas MiniHaps with ≥5 SNPs require long-read sequencing for accurate haplotype reconstruction. This phasing dependency defines the analytical boundary between the two marker classes [29,74]. A 22-MiniHap panel achieved a mean Ae of 10.96 (compared with 3–5 for standard MHs), with 52% of loci exceeding Ae 12.0 and a combined match probability of 4.45 × 10^−31^ [29]. Mixture analysis demonstrated detection limits of 1:39 in two-person mixtures and 1:8:1 for three-person mixtures [29]. These findings illustrate a broader trend toward increasing per-locus information content using next-generation sequencing platforms.

### 3.7. Marker Strategy Summary

Novel marker systems reduce allele sharing, increase per-locus information content, and improve resistance to DNA degradation, thereby decreasing analytical complexity at the molecular level rather than relying solely on downstream algorithmic refinement. In practical terms, these properties enhance deconvolution robustness across complex and degraded samples. However, translating molecular advantages into routine forensic practice requires structural support. Broader implementation will depend on the establishment of standardized locus panels analogous to CODIS core loci, development of validated analytical pipelines compatible with continuous probabilistic genotyping frameworks, and harmonization of deconvolution-specific performance metrics.

## 4. Probabilistic Genotyping: Algorithmic Solutions

When physical separation and high-information markers are insufficient to resolve a DNA mixture, probabilistic genotyping (PG) extracts information directly from quantitative features of electropherograms or sequencing read data. This section reviews the evolution from qualitative to fully continuous models, compares major platforms, discusses extensions to non-STR markers, and examines implications for genotype reconstruction and LR evaluation.

### 4.1. From Qualitative to Fully Continuous Models

Early semi-continuous models (e.g., LRmix Studio) reduced electropherograms to binary allele presence/absence states, incorporating drop-out and drop-in while discarding peak height information [75,76]. Comparative analyses demonstrated lower true-positive rates relative to fully continuous models [3]. Fully continuous models incorporate peak height (or read depth) directly into the likelihood function. STRmix™ applies a log-normal peak height model [4], whereas EuroForMix and DNAStatistX employ gamma distributions [3,77]. By modeling quantitative signal variation, continuous systems better accommodate allele sharing and stochastic effects in complex mixtures.

### 4.2. Mainstream PG Platforms

#### 4.2.1. STR-Based Systems

The most widely deployed STR-based PG platforms include STRmix™, EuroForMix, TrueAllele™, and Statistefix 4.0 (Table 2). Although differing in statistical architecture, all implement continuous modeling frameworks and have undergone developmental and/or casework validation.

STRmix™ uses Bayesian Markov chain Monte Carlo (MCMC) sampling with Metropolis–Hastings algorithms [4]. A 31-laboratory internal validation study encompassing 2825 mixtures reported consistent LR performance across kits, instruments, and mixture ratios [79]. Version 2.6 introduced support for treating the number of contributors (NOC) as a nuisance parameter for two consecutive values [3], and an NGS implementation incorporating sequence-based stutter models has completed developmental validation [89]. Key concerns involve the proprietary codebase; the Federal Judicial Center has highlighted the importance of third-party source-code review [91].

EuroForMix, the first open-source fully continuous system [93], has demonstrated close agreement with STRmix™ in comparative studies: single-source LRs matched to four significant figures, and mixture LRs generally differed within one order of magnitude [80]. The EFMrep extension supports joint analysis of samples amplified with different kits and allows specification of pairwise kinship among unknown contributors [90].

TrueAllele™ employs a hierarchical Bayesian model in which contributor genotypes, mixture proportions, and nuisance parameters are jointly inferred via MCMC [78,81]. A distinguishing feature is reference-free genotype inference. Contributor genotypes are estimated prior to comparison with any suspect profile [81,84]. The system supports iterative conditioning (“genotype peeling”) to refine estimates when known contributors are incorporated [84]. The system includes a built-in probabilistic genotype database for automated direct and familial searches without external modules [85]. Validation studies have included laboratory mixtures with up to ten unknown contributors [84], 368 adjudicated New York State Police casework items [81], and 72 Virginia criminal cases [82], with reported false positive rate below 0.005%. An independent validation by the Virginia Department of Forensic Science confirmed performance in 2–4 contributor mixtures [83]. Like STRmix™, TrueAllele is proprietary and has faced legal challenges regarding source code disclosure (notably State v. Pickett, 2021 [92]); the PCAST report (2016) recommended additional independent validation studies [94].

Statistefix 4.0 is a freely available automated tool designed for high-throughput screening [77]. In a three-laboratory study including 2626 reference samples and 7662 casework samples, major-contributor identification performance was comparable to established systems, although allele uncertainty was higher. It is primarily suited for triage workflows preceding full continuous analysis. Accordingly, its published validation scope is narrower than that of the other three platforms, consistent with its role as a screening and triage tool rather than a comprehensive probabilistic genotyping system.

Although optimized for LR calculation, these systems also generate probabilistic genotype outputs. Such outputs are influenced by NOC specification, mixture proportions, and template quantity [95], and therefore warrant independent consideration when used for genotype reconstruction.

#### 4.2.2. Extension to Non-STR Markers

Continuous PG principles are not restricted to STR data.

##### MHs

MH-specific continuous models have recently been developed. A truncated Gaussian (TG) model for MH read counts reported major contributor deconvolution accuracy of 0.9145 across 90 mixtures [96]. Comparative analyses indicated improved performance of MH panel over STR kit for two-person mixtures, although per-locus polymorphism can limit resolution in highly complex scenarios [97]. Existing PG platforms such as EuroForMix have been adapted to process MH read coverage data, with stutter modeling disabled [96,97].

##### SNP Mixtures

EuroForMix has also been applied to complex SNP mixture analysis [98]. Studies combining 94 iiSNPs with 27 STRs using ForenSeq™ reported correct minor-donor assignment rates approaching 98% [99]. For forensic genetic genealogy, MixDeR integrates SNP deconvolution with EuroForMix and formats inferred profiles for GEDmatch^®^ PRO [52]. Such workflows enable identity-by-descent-based relative searching beyond the scope of STR-only approaches [55,100].

### 4.3. Algorithmic Advances in PG Inference

Recent methodological developments have focused on improving posterior stability, computational efficiency, and genotype reconstruction accuracy.

#### 4.3.1. Hamiltonian Monte Carlo (HMC)

MCMC-based systems exhibit run-to-run variability. Implementation of Hamiltonian Monte Carlo (HMC) with strict convergence diagnostics reduced log_10_LR variability by approximately tenfold without increasing runtime [101]. While primarily evaluated in LR terms, improved posterior stability is expected to enhance reproducibility of genotype estimates.

#### 4.3.2. Variational Inference (VI)

Variational inference (VI) methods, including Stein Variational Gradient Descent (SVGD), have achieved approximately 4.3-fold acceleration compared with standard MCMC while maintaining comparable LR precision on PROVEDIt datasets [102]. Such acceleration is particularly relevant for database-searching workflows and large-scale validation studies.

It should be noted that both HMC and VI have been evaluated primarily in terms of LR precision and computational efficiency. Genotype-level reconstruction accuracy—the metric most directly relevant to the deconvolution objective of this review—has not yet been systematically reported for these methods. By contrast, the deep-learning approach of Yu et al. (2025) [103] represents a notable exception in directly evaluating genotype concordance. This disparity reflects the broader gap identified in Section 4.4.1: most algorithmic advances continue to be assessed using LR-centric criteria.

#### 4.3.3. Deep Learning-Enabled Deconvolution

A ResNet-based locus-dependency model trained on single-source STR profiles has been used to re-weight genotype probabilities from a continuous engine [103]. On PROVEDIt mixtures, genotype reconstruction accuracy improved by up to 30 percentage-points relative to the baseline continuous model. Performance was evaluated directly using genotype concordance rather than LR thresholds, highlighting the potential of inter-locus modeling to improve deconvolution fidelity. In parallel, artificial neural network–based electropherogram peak classification has been integrated with STRmix™ to enable automated “lights-out” workflows [104], reducing analyst intervention without altering the underlying generative model.

### 4.4. Deconvolution and LR Evaluation: Distinct Objectives

**Figure 2:**
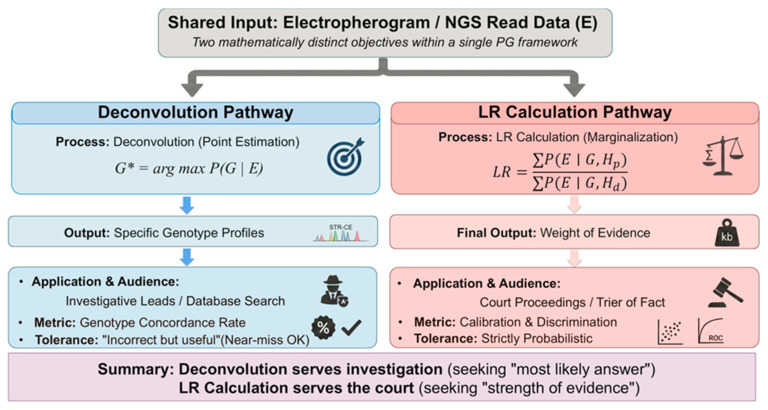
**Deconvolution and LR evaluation as distinct inferential objectives within PG frameworks.** Both tasks operate on identical input data but differ in mathematical formulation, output structure, acceptable error profiles, and validation metrics. Deconvolution prioritizes genotype reconstruction fidelity, whereas LR evaluation emphasizes hypothesis discrimination and evidential calibration.

PG software addresses two mathematically distinct tasks within a unified inferential framework: identifying contributors and quantifying evidential strength (Figure 2). Deconvolution seeks to reconstruct individual contributor genotypes---whether as a point estimate G* = argmax P(G|E), a ranked set of candidate genotypes, or a full posterior distribution P(G|E)—whereas LR calculation marginalizes over all genotype sets under competing hypotheses to quantify evidential weight.

Although these objectives share computational machinery, they differ fundamentally in purpose, error tolerance, and validation criteria. Deconvolution prioritizes genotype reconstruction fidelity, particularly in investigative contexts, whereas LR evaluation emphasizes calibration and discrimination between hypotheses.

Architecturally, STRmix™ and EuroForMix require analyst-specified NOC, whereas TrueAllele™ estimates contributor number empirically from data [84], as described in the NIST Scientific Foundation Review [5]. The systems further differ in peak height distributions, stutter parameterization, convergence diagnostics, and threshold handling. These modeling differences can propagate into divergent posterior genotype distributions—and consequently divergent LR outputs—from identical input data.

Thompson (2023) highlighted this sensitivity in a federal case analysis in which STRmix™ reported an LR of 24 in favor of the non-contributor hypothesis, while TrueAllele™ reported values between 1.2 million and 16.7 million for the same evidence [105]. Locus-by-locus evaluation attributed the divergence to differences in modeling parameters, analytic thresholds, and mixture proportion estimation. Importantly, such discrepancies do not necessarily imply analytical error by either system; rather, they illustrate how defensible but non-identical modeling assumptions can substantially influence LR magnitude. The case underscores that PG outputs are conditional on model structure and parameterization, and therefore require transparent documentation and case-type-matched validation. Current validation guidelines, including the SWGDAM Guidelines for the Validation of Probabilistic Genotyping Systems (2015) [106] and the ISFG DNA Commission recommendations [107], focus on establishing each system’s internal reliability but do not prescribe procedures for reconciling divergent outputs when multiple PG systems are applied to the same evidence. In practice, most forensic laboratories use a single validated system, so inter-system comparison rarely arises in routine casework. However, as PG tools become more widely adopted and defense experts increasingly conduct independent analyses, the need for guidance on interpreting and reporting inter-system discrepancies may become more pressing. The Thompson (2023) case illustrates that such divergence can be traced to identifiable modeling differences rather than arbitrary inconsistency, but transparent reporting of analytical parameters and assumptions is essential for judicial evaluation.

More broadly, the admissibility of PG tools varies across jurisdictions. In the United States, the Daubert standard focuses on methodology and error rates, while other jurisdictions apply different thresholds for expert scientific evidence. The 2016 PCAST report and subsequent court decisions (e.g., State v. Pickett, 2021) have shaped the evolving landscape of PG admissibility, particularly regarding whether independent validation of proprietary systems is sufficient [92,94]. The tension between intellectual property protections and defendants’ rights to examine evidence remains unresolved: the Federal Judicial Center has emphasized the importance of source-code review [91], yet proprietary codebases in STRmix™ and TrueAllele™ limit independent scrutiny. For forensic genetic genealogy (FIGG), the U.S. Department of Justice interim policy (2019, updated 2023) provides guidance on consent and investigative scope, but ethical standards for genealogy database searching continue to evolve across jurisdictions.

#### 4.4.1. The Need for Standardized Deconvolution Metrics

The absence of standardized performance metrics for genotype reconstruction complicates cross-platform comparison. Current studies report heterogeneous outcome measures, including:

(i) Per-locus genotype error rates (e.g., MHs vs. STR comparisons in Section 3.4.1);
(ii) Overall genotype concordance proportions (e.g., 0.9145 for the MHs continuous model [96]; 41.1–57.5% for deep-learning deconvolution [103]);
(iii) LR-derived sensitivity and specificity at selected thresholds (e.g., LR > 1 for contributor detection in Section 4.2.2; LR > 10^6^ for database searching in Section 5.1);
(iv) False positive rates against known non-contributors (e.g., <0.005% in TrueAllele casework validation).

These metrics are not directly comparable. A system may achieve strong LR sensitivity while recovering incorrect alleles at multiple loci or conversely exhibit high genotype concordance but modest LR magnitude under conservative modeling assumptions. Without unified benchmarks—analogous to the sensitivity, specificity, and precision standards articulated by SWGDAM for LR validation [106] and the ISFG DNA Commission recommendations [107]—it remains difficult to systematically evaluate deconvolution fidelity across platforms. The absence of genotype-level validation criteria represents a structural gap in current PG evaluation frameworks and may hinder development of algorithms optimized specifically for genotype reconstruction.

#### 4.4.2. The Case for Deconvolution-Specific Algorithms

Current PG systems primarily optimize LR calculation; genotype posterior distributions arise as intermediate products of hypotheses testing rather than as directly optimized outputs. In this sense, deconvolution remains embedded within LR-centric architectures. TrueAllele™ approaches a deconvolution-oriented design through reference-free genotype separation and built-in database functionality. Nevertheless, its genotype inference remains part of a unified Bayesian framework constructed for evidential comparison, and NOC estimation—although data-driven—is not formalized as an explicit model-selection framework with defined penalties for over-parameterization.

A fully deconvolution-specific architecture would extend beyond current implementations in several respects:

(i) Simultaneous NOC inference—treating NOC as a model-selection problem rather than a fixed or analyst-guided input, given the substantial impact of NOC misspecification on genotype reconstruction accuracy.
(ii) Multimodal posterior reporting—explicitly presenting multiple high-probability genotype solutions rather than reducing inference to a single “most probable” estimate, particularly in allele-sharing scenarios where several genotype combinations may explain the observed data nearly equally well.
(iii) Optimized mixture proportion estimation—since genotype reconstruction accuracy, especially for minor contributors, is highly sensitive to mixture ratio precision, even when LR magnitude remains comparatively stable.
(iv) Marker-specific noise models—differentiating between STR stutter, NGS sequencing error, and dropout patterns rather than relying on uniform noise assumptions.
(v) Inter-locus dependency modeling—as demonstrated by Yu et al. (2025), modeling between-locus corrections improved genotype reconstruction accuracy by up to 30 percentage points, suggesting that locus-independence assumptions leave recoverable information unused [103].

Of the five features outlined above, simultaneous NOC inference (i) and marker-specific noise models (iv) reflect formalization of challenges already recognized in the literature, while multimodal posterior reporting (ii) and optimized mixture proportion estimation (iii) extend existing concepts into explicitly deconvolution-oriented design criteria. Inter-locus dependency modeling (v) represents the most novel proposal, supported by recent empirical demonstration [103]. The two-stage architecture itself—separating genotype reconstruction from LR evaluation as independently optimized and validated modules—represents the principal original contribution of this section.

Operationally, such an approach could adopt a two-stage architecture: an initial module optimized for genotype reconstruction fidelity, followed by a separate LR module for evidential evaluation. Each stage could then be validated using metrics appropriate to its objective—genotype concordance for deconvolution and calibration/discrimination metrics for LR. This separation would also facilitate downstream integration with STR database searching (DBLR™, ProbRank), genealogical pipelines (MixDeR, GEDmatch), and prospective SNP-to-STR imputation workflows.

### 4.5. Summary of Section 4

Fully continuous PG systems have transformed mixture interpretation into a quantitative modeled discipline, and their extension to MH and SNP panels has broadened investigative applications. Algorithmic developments—including HMC (approximately tenfold reduction in run-to-run), VI (approximately 4.3-fold acceleration), and deep-learning-assisted locus modeling (up to ~30 percentage-point gains in genotype concordance)—indicate that both statistical and machine-learning approaches can improve inferential stability and reconstruction fidelity.

At the same time, comparative case analyses demonstrate that LR outputs remain sensitive to modeling assumptions. While major PG systems have achieved substantial courtroom acceptance, two structural tensions persist: proprietary codebases limit independent scrutiny, and existing validation guidelines focus primarily on LR calibration without specifying genotype-level reconstruction benchmarks.

As deconvolution becomes increasingly relevant for investigative workflows and database integration, future validation frameworks will need to incorporate standardized genotype concordance metrics alongside traditional LR performance measures.

## 5. Maximizing the Application of Deconvolution Results: Evolution of Database Searching Strategies

Database integration strategies are at an earlier stage of empirical validation compared to the molecular and algorithmic approaches discussed in Section 2, Section 3 and Section 4. This section focuses on key proof-of-concept demonstrations, with references to the original validation studies for detailed methodology. The practical value of mixture deconvolution lies in converting probabilistic genotype outputs into actionable investigative leads. However, PG outputs are not deterministic genotype profiles; rather, they consist of probability distributions over possible genotype sets, often with residual uncertainty. Bridging the gap between probabilistic inference and existing database infrastructure therefore represents a critical translational challenge. Three principal strategies have emerged, reflecting different balances between statistical rigor, computational demand, and compatibility with legacy database systems.

### 5.1. LR-Based Direct Searching

The most statistically coherent strategy is direct LR-based searching, in which mixture evidence is compared against database profiles within the same probabilistic framework used for casework interpretation.

DBLR™ (STRmix™-based) enables rapid computation of LRs across large databases, supporting direct searching, mixture-to-mixture comparison, and kinship evaluation [86]. CaseSolver (EuroForMix-based) implements a staged screening approach, progressing from allele filtering to full quantitative evaluation [87]. ProbRank (DNAStatistX-based) computes quantitative LRs directly from mixture evidence without prior deterministic deconvolution and has demonstrated improved retrieval of minor contributors relative to qualitative ranking approaches [88].

Empirical validation supports the scalability of LR-based searching. Nozownik et al. (2025) searched 40 prepared mixtures (2–5 contributors) against the Swiss National DNA Database (174,493 individual profiles) using DBLR™ [108]. With LR thresholds of 10^3^ and 10^6^, sensitivity/specificity were 90.0%/99.9% and 57.1%/100.0%, respectively. At the lower threshold, this resulted in only 52 adventitious associations across more than 24 million pairwise comparisons. In a subsequent casework phase involving 160 mixtures (2–4 contributors), LR-based searching identified 380 associations, including 186 new investigative leads not recovered by prior local comparison workflows [108]. Complementarily, Taylor et al. (2021) validated a “top-down” STRmix™ workflow designed to prioritize major contributors in complex mixtures [109]. Applied to 91 no-suspect casework samples, approximately 75% produced database links, corresponding to an estimated 83 additional investigative leads annually within a single laboratory. Together, these findings indicate that LR-based searching can convert mixtures previously considered unsuitable for database comparison into productive investigative resources.

### 5.2. Translating Probabilistic Outputs into Legacy Infrastructure

While LR-based direct searching is statistically optimal, many jurisdictions continue to operate within CODIS-style deterministic database infrastructures. Transitional strategies therefore aim to translate probabilistic genotype outputs into bounded deterministic candidate sets.

One approach categorizes loci into high-confidence and low-confidence genotype states based on posterior probabilities, then generates a finite set of candidate profiles from uncertain loci for conventional database submission. This method preserves compatibility with existing search engines while limiting combinatorial explosion.

SmartRank represents an early validated implementation of such a transitional framework. Using LRmix-derived likelihood ratios incorporating dropout and drop-in modeling, SmartRank ranks database candidates above defined LR thresholds [110]. Validation across 343 mixed DNA profiles and over 750 searches demonstrated improved recovery of true contributors relative to traditional allele-count matching.

Building on this concept, the Netherlands Forensic Institute implemented an automated workflow (Fast ID Line v2.0) integrating machine-learning–based NOC estimation with quantitative database searching [111]. Compared with earlier qualitative approaches, the updated system retrieved nearly twice as many candidate associations (304 vs. 162 out of 777 searches) while maintaining rapid turnaround times.

These transitional systems illustrate how probabilistic inference can be incrementally integrated into legacy infrastructures without requiring immediate nationwide database redesign.

### 5.3. Probability-Weighted Similarity Approaches

An alternative strategy assigns similarity scores to database profiles based on concordance with PG-derived allele probability distributions. Candidates are ranked according to aggregate weighted scores rather than full LR computation. Such approaches offer computational efficiency and tolerance for uncertainty; however, weighted scores lack the formal probabilistic interpretability and cross-laboratory standardization of LR-based methods. Accordingly, they are best considered complementary tools for investigative triage rather than substitutes for fully probabilistic searching.

### 5.4. Summary of Section 5

LR-based direct database searching is increasingly emerging as the most statistically coherent strategy for mixture comparison. Empirical evaluations have reported sensitivities and specificities approaching 57–90% depending on LR threshold, with near-perfect specificity, while recovering investigative leads that would previously have remained undetected. Candidate enumeration remains a pragmatic interim solution within the existing CODIS infrastructure, and probability-weighted similarity approaches provide additional operational flexibility under resource or infrastructure constraints.

Looking ahead, the convergence of these strategies with automated “lights-out” workflows—integrating neural network–based electropherogram interpretation, fully continuous probabilistic genotyping, and LR-based database comparison within a unified computational pipeline (e.g., the FaSTR™ DNA/STRmix™/DBLR™)—suggests a progressive shift toward highly automated forensic DNA processing. As demonstrated by Nozownik et al. (2025), PG-driven database searching can convert mixtures previously considered too complex for database use into productive investigative leads, thereby expanding the operational utility of forensic DNA in complex casework [108].

## 6. Conclusions and Prospects

This review examined DNA mixture deconvolution through a four-strategy framework encompassing physical separation, high-information genetic markers, PG algorithms, and database integration. Rather than representing competing approaches, these strategies operate at different analytical layers: upstream molecular simplification reduces inferential burden, while downstream computational modeling extracts maximal information from residual overlap.

A central theme of this review is that genotype reconstruction and LR evaluation, although often implemented within the same probabilistic genotyping framework, constitute distinct inferential objectives. Current PG systems were primarily developed for LR calculation and evidential reporting; genotype reconstruction typically arises as an intermediate output. As deconvolution becomes increasingly relevant in investigative and database-search contexts, explicit validation of genotype-level performance metrics may become necessary alongside traditional LR-based standards.

The convergence of physical separation technologies (e.g., single-cell isolation and sequencing), high-information marker systems (e.g., microhaplotypes and MiniHaps), and advances in statistical and machine-learning inference is progressively narrowing the historical information gap in mixture interpretation. At the same time, integration with LR-based database infrastructures is expanding the operational scope of complex mixture analysis.

Looking forward, several priorities merit attention: 1. Establishment of standardized MH locus panels through coordinated international efforts, analogous to the CODIS core loci. 2. Development of hybrid NGS panels enabling simultaneous STR, SNP, and MH typing within unified workflows. 3. Design and validation of deconvolution-oriented algorithms using genotype concordance and reconstruction accuracy as primary benchmarks. 4. Expansion of national database infrastructures to support LR-based direct searching. 5. Formulation of case-type-specific best-practice guidelines for selecting among molecular and computational strategies. The increasing reliance on PG-derived evidence in criminal proceedings, combined with the rapid expansion of FIGG as an investigative tool, lends practical urgency to these priorities. Without standardized deconvolution benchmarks, transparent validation frameworks, and interoperable database infrastructure, the gap between algorithmic capability and operational accountability will continue to widen.

This review focuses on nuclear DNA-based genotype reconstruction for individual identification. Topics intentionally excluded or only briefly addressed include mitochondrial DNA mixture analysis in depth, X-chromosomal markers, epigenetic profiling, and population-specific reference panel optimization. As a narrative review, the literature selection may be influenced by publication bias toward positive or novel results. The review is based predominantly on English-language publications; relevant work in other languages may not be fully represented. The field is evolving rapidly, and several tools described here were published in 2025–2026 and may be updated or superseded. Readers are encouraged to consult current software documentation and validation studies.

Rather than a single universal solution, the field appears to be moving toward context-dependent integration of molecular innovation and probabilistic modeling. Continued alignment between methodological development, validation standards, and operational practice will determine how effectively DNA mixture deconvolution evolves from a technically challenging inference problem into a routinely deployable investigative tool.

## Figures and Tables

**Table 1:** Comparison of Genetic Markers for DNA Mixture Deconvolution.

Marker Type	Core Characteristics	Deconvolution Advantages	Limitations/Challenges	Validated Mixture Complexity	Optimal Application Scenarios	Representative Technology/Panel
**CE-STR**	Length polymorphism; 10–30 core loci; long amplicons (>200 bp)	Mature and standardized; global CODIS databases; established PG software support	Severe stutter artifacts; amplification imbalance; sensitive to degradation and low-template DNA; allele sharing limits deconvolution in balanced mixtures	Routine: 2-person; challenging: 3–4 person	Routine single-source or simple two-person mixtures; mandatory for CODIS searches	PowerPlex^®^ Fusion; GlobalFiler; Investigator^®^ 24plex QS Kit
**NGS-STR**	Sequence-level polymorphism at STR loci; detects 23–30% more alleles than CE-STR [22,23,24,25]	Higher discrimination than CE-STR; distinguishes length-identical alleles by internal sequence; short amplicons (<150 bp) improve degraded DNA performance	Sequence stutter persists; NGS-specific artifacts such as intra-locus noise, and length-dependent amplification imbalance, in addition to the higher computational demands higher cost and more complex data analysis than CE-STR	2–3 person (improved minor-contributor detection at <5% ratio)	Cases requiring higher discrimination; complement to CE-STR in complex mixtures	ForenSeq™ DNA Signature Prep Kit; Precision ID GlobalFiler™ NGS STR Panel v2; PowerSeq^®^ 46GY System
**Microhaplotypes (MHs)**	2–6 tightly linked SNPs; short amplicon (<300 bp); length-invariant alleles; no stutter	96.4% contributor-specific alleles vs. 51.3% for CE-STR [26]; excellent heterozygote balance; degradation-resistant; 4–5× lower genotype error rates than STR deconvolution [27]	No global standard panels yet; per-locus Ae may be lower than STRs; requires NGS infrastructure and specialized PG models	2–5 person (163-plex panel) [28]	Complex (≥3 person) or balanced mixtures; moderately degraded or low-template DNA	Ion AmpliSeq™ MH-74 Plex; custom panels (87-, 124-, 163-plex)
**Mini-haplotype (MiniHap)**	Haplotypes with ≥5 SNPs; requires long-read sequencing for accurate phasing	Ultra-high polymorphism (average Ae = 10.96 vs. 3–5 for standard MHs); minor-contributor detection at 1:39; combined match probability 4.45 × 10^−31^ [29]	Proof-of-concept stage; requires nanopore or other long-read platforms; cross-population validation needed	2-person (1:39); 3-person (1:8:1) [29]	Future ultra-complex mixture analysis requiring maximum per-locus information	Research panels (22-MiniHap panel via nanopore sequencing)
**DIP-STR**	Composite: DIP + adjacent STR; allele-specific amplification	Exceptional sensitivity for extremely unbalanced 2-person mixtures (up to 1:1000); selective amplification of 0.03–0.1 ng minor DNA [30,31]	Limited to 2-person mixtures; STR stutter effects persist; requires specialized primer design	2-person (up to 1:1000 ratio) [32]	Sexual assault cases (sperm/epithelial); trace contributors in touch DNA	Validated panels (10-plex, 23-plex)

**Table 2:** Comparison of STR-Based Probabilistic Genotyping Platforms.

Feature	STRmix™	EuroForMix	TrueAllele™	Statistefix 4.0
**Statistical framework**	Bayesian MCMC (Metropolis–Hastings); log-normal peak height model [4]	Maximum likelihood estimation; optional Bayesian mode; gamma peak height model [77]	Bayesian MCMC; hierarchical continuous model [78]	Automated MLE; continuous model
**License**	Commercial (closed-source)	Open-source (R package)	Commercial (closed-source)	Free
**Key validation Studies**	31 labs, 2825 mixtures [79]; LR agreement with EuroForMix [80]	LRs within 1 order of magnitude of STRmix™ [80]; widely adopted across European labs	368 casework items (NYSP) [81]; 72 Virginia cases [82]; independent Virginia DFS validation [83]	3 labs; 2626 references + 7662 casework samples [77]
**NOC**	Routinely 2–4 (versions ≤v2.10); v2.11+ routinely supports 5-person mixtures; v2.6 supports NOC-as-nuisance for two consecutive values [3]	≤4 unknown contributors; runtime increases substantially beyond 3	Up to 10 unknown contributors on laboratory-prepared mixtures [84]	Primarily validated for major-contributor identification
**NOC handling**	Analyst-specified; NOC-as-nuisance option [3]	Analyst-specified	Empirically estimated from data; analyst can override [84]	Analyst-specified
**Deconvolution output**	Posterior genotype distributions; MAP genotype for DBLR™ searching	Posterior genotype probabilities per contributor	Reference-free probabilistic genotypes stored in built-in database [81,85]	Automated major-contributor calls
**Database searching**	Via DBLR™ [86]	Via CaseSolver [87]	Built-in TrueAllele Database; automated direct and familial searching [85]	Via ProbRank [88]
**NGS compatibility**	Yes; NGS version with sequence-based stutter models validated [89]	Yes; processes MHs and SNP read-count data; stutter modeling disabled for MH loci	Not explicitly validated for NGS in published literature	Not reported
**Strengths**	Broad court acceptance; large-scale multi-lab validation; NGS version available; NOC-as-nuisance feature	Open-source transparency; EFMrep extension for joint kit analysis and kinship [90]; active community development	Fully automated (no analyst thresholds); reference-free genotype separation; built-in database searching; WTC disaster identification	Free access; automated batch screening; rapid triage of large sample volumes
**Limitations**	Proprietary codebase limits independent scrutiny; source-code review recommended by Federal Judicial Center [91]	Runtime ceiling for >4 unknowns; less extensive court acceptance history	Proprietary codebase; most validations by developer; source code access contested in courts [92]	Higher allele uncertainty vs. established platforms [77]; limited validation scope

## Data Availability

No new data were created or analyzed in this study.

## Abbreviations

The following abbreviations are used in this manuscript:

Ae	Effective allele number
CE-STR	Capillary electrophoresis-based short tandem repeat
DBLR	Database Likelihood Ratio
DIP	Deletion/insertion polymorphism
DSCS	Direct single-cell subsampling
FIGG	Forensic investigative genetic genealogy
HMC	Hamiltonian Monte Carlo
iiSNP	Identity-informative single nucleotide polymorphism
LR	Likelihood ratio
MAP	Maximum a posteriori
MCMC	Markov chain Monte Carlo
MH	Microhaplotype
MiniHap	Mini-haplotype
MLE	Maximum likelihood estimation
MNP	Multi-SNP
NGS	Next-generation sequencing
NOC	Number of contributors
PG	Probabilistic genotyping
scDNA-seq	Single-cell DNA sequencing
SNP	Single nucleotide polymorphism
STR	Short tandem repeat
VI	Variational inference
WGS	Whole-genome sequencing
